# Acute leaflet malfunction of Navitor valve with severe intraprosthetic aortic insufficiency immediately after implantation

**DOI:** 10.1007/s12928-024-00995-6

**Published:** 2024-03-29

**Authors:** Ryo Horita, Daisuke Hachinohe, Ryo Otake, Juan Armando Diaz, Hidemasa Shitan, Tsutomu Fujita

**Affiliations:** 1 Department of Cardiology, Asia Medical Group, Sapporo Heart Center, Sapporo Cardio Vascular Clinic, North 49, East 16, 8-1, Higashi Ward, Sapporo, Hokkaido 007-0849 Japan; 2 Makati Medical Center, Makati, Philippines

**Keywords:** Aortic valve stenosis, Navitor, Intraprosthetic aortic insufficiency, TAV-in-TAV

**Fig. 1:**
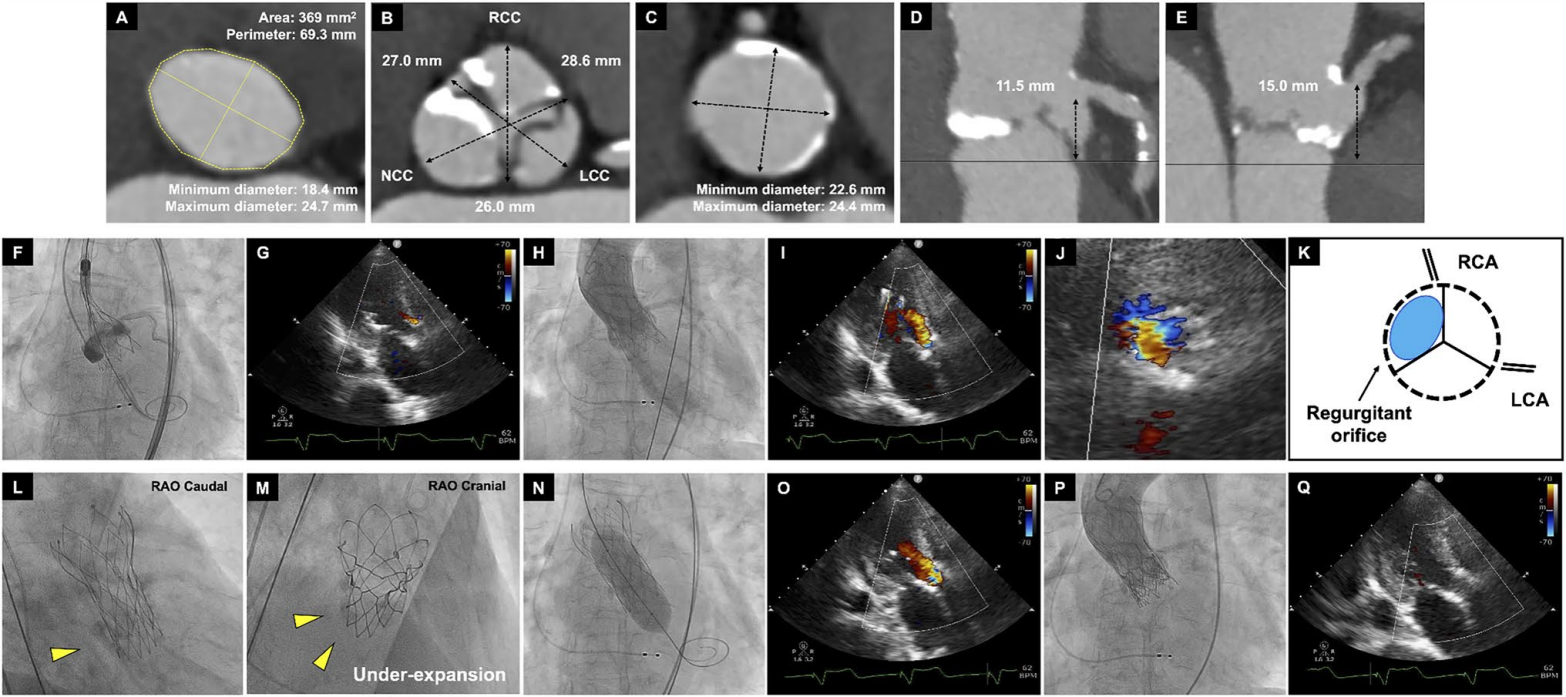
**A**–**E** Preoperative CT measurement match to 25 mm Navitor. **A** Aortic annulus, **B** sinus of Valsalva, **C** sinotubular junction, **D, E** left/right coronary heights. **F** Implantation depth was about 4 mm in fluoroscopy.** G** TTE revealed mild PVL at point of no recapture. **H**, **I** Acute severe intraprosthetic insufficiency occurred and required inotropes to maintain blood pressure. **J** TTE short axis view of diastolic phase showed regurgitant orifice at non coronary cusp (NCC) site. **K** Schema of TTE short axis view. **L**, **M** RAO Caudal and RAO Cranial view revealed under-expansion of Navitor at NCC site. **N**, **O** After post dilatation with 20 mm balloon, regurgitation became worse. **P** 20 mm S3UR in 25 mm Navitor was performed under left coronary protection. **Q** TTE showed no PVL and optimal prosthetic function

An 85-year-old female was admitted our hospital due to congestive heart failure secondary to very severe aortic stenosis. Preoperative computed tomography (CT) demonstrated severe leaflet calcification and anatomical measurements confirmed suitability for transcatheter aortic valve (TAV) implantation (Fig. 1A–E), using a 25 mm Navitor (Abbott, Abbott Park, IL, USA). After pre-dilatation with an 18 mm balloon, a 25 mm Navitor was deployed. The Implantation depth was acceptable and transthoracic echocardiography (TTE) revealed mild paravalvular leakage (PVL) at the point of no recapture (Fig. 1F and G).

Immediately after deployment, acute severe intraprosthetic regurgitation occurred (Fig. 1H and I, Video 1).

TTE short axis view revealed regurgitant orifice at non coronary cusp (NCC) site (Fig. 1J and K, Video 2) and RAO Caudal and RAO Cranial view also demonstrated under-expansion of Navitor at NCC site due to asymmetrical severe calcification (Fig. 1L and M). These two findings indicated that under expansion at NCC site resulted in leaflet malfunction. Post-dilatation was attempted with a 20 mm balloon, but regurgitation notably worsened (Fig. 1N and O). Finally, TAV-in-TAV using a 20 mm SAPIEN 3 Ultra RESILIA (S3UR Edwards Lifesciences, Irvine, CA, USA) with overfilling inflation to make its stent frame height shorter and overhang the Navitor’s leaflet was performed under left coronary protection (Fig. 1P, Videos 3 and 4). Despite potential high risk of coronary obstruction (CO) due to a narrow sinotubular junction (STJ), CO did not occur during the procedure. Intraprosthetic regurgitation resolved and prosthetic function appeared optimal on TTE (Fig. 1Q).

To our knowledge, this is the first reported case of acute severe intraprosthetic insufficiency immediately after Navitor implantation requiring TAV-in-TAV using S3UR. Several articles have reported this phenomenon following SAPIEN valve implantation, with some instances requiring TAV-in-TAV [1]. The stuck leaflet is suspected to be the cause, but it remains unclear. While post-dilatation worsened regurgitation and CT revealed potential high risk of CO, S3UR-in-Navitor resulted in a successful outcome in this case.

### Supplementary Information

Below is the link to the electronic supplementary material.

Supplementary file1 Severe intraprosthetic insufficiency occurred (MP4 6855 KB)

Supplementary file2 TTE short axis view demonstrated eccentric intraprosthetic regurgitation from NCC site (MP4 3630 KB)

Supplementary file3 20 mm S3UR was successfully implanted in Navitor (MP4 20437 KB)

Supplementary file4 Final aortography confirmed no intraprosthetic insufficiency (MP4 10044 KB)
